# Health care seeking behavior among presumptive tuberculosis patients in Ethiopia: a systematic review and meta-analysis

**DOI:** 10.1186/s12913-020-05284-5

**Published:** 2020-05-19

**Authors:** Dinka Fikadu Gamtesa, Habteyes Hailu Tola, Zemedu Mehamed, Ephrem Tesfaye, Ayinalem Alemu

**Affiliations:** Ethiopian Public Health Institute, Tuberculosis/HIV Research Directorate, PO Box 1242, Addis Ababa, Ethiopia

**Keywords:** Health seeking, Health seeking behavior, Health facility

## Abstract

**Background:**

Health seeking behavior is one of the challenges affecting tuberculosis (TB) control program because of its high risk to prolonged diseases transmission and poor treatment outcome. Although there are few primary studies that reported diversified magnitudes of health seeking behavior among presumptive TB patients in Ethiopia, there is no review study that attempted to summarize the available evidence. Thus, this review was aimed to estimate the proportion of health care seeking behavior from health facility and to summarize the reasons why individuals with presumptive TB are not seeking health care in Ethiopia.

**Method:**

A systematic review and meta-analysis study was conducted on primary studies that reported proportion of health seeking behavior among presumptive TB patients. Electronic databases: PubMed, Google Scholar and Science Direct were searched to retrieve studies published in English language from Ethiopia without restricting publication year. In addition, bibliographies of included studies were also screened to retrieve potential studies. The keywords “health seeking”, “health seeking behavior”, “TB suspects” and “presumptive TB” were used both in Medical Subject Heading (MeSH) and free text. Random effects meta-analysis model was used to estimate the pooled proportions of health care seeking and not seeking behaviors. Stata version 14 was used for data analysis.

**Result:**

Five studies which involved 3230 patients with presumptive TB were included into this review. The pooled estimated proportion of health care seeking behavior among presumptive TB patients from health facilities was 65% (95% CI, 54–76%), while the pooled proportion of not seeking health care from any sources was 17% (95% CI;6–27%). In addition, 18% (95% CI; 5–30%) of presumptive TB patients were seeking health care from inappropriate sources. Being female, younger age, low income status, absence of previous TB treatment history, low education status were the risk factors that associated with low health care seeking behavior.

**Conclusion:**

Considerable proportion of patients with presumptive TB were not seeking health care from health facilities or seeks care from inappropriate sources in Ethiopia. Implementing efforts that could improve health care seeking behavior is vital to prevent prolonged disease transmission through immediate treatment commencement.

## Background

Tuberculosis (TB) is an infectious disease that affects millions of people each year across the world [1]. It causes 1.8 million deaths and 10.4 million new cases in 2018 globally [1]. It is also one of the top 10 causes of death and leading cause of death from a single infectious agent [1, 2].

Health care seeking behavior in presumptive TB patients is defined as how patients with presumptive TB in the community seek help from nearby health facility or utilize health care for their TB symptoms to attain or regain good health and to prevent illness [3]. Presumptive TB is also defined as a patient who presents with the signs and symptoms suggestive of TB [4]. Health facilities in this review include health post, health center and hospitals at different levels and private health institutions that have legal license to diagnose and treat TB patients. Inappropriate health care sources include traditional healers, holly water, drug store and witchcrafts that has no license to diagnose and treat TB.

Not seeking or delay in seeking health care from health facilities are the potential risk of prolonged disease transmission and lead to poor treatment outcome [3, 5]. For these reason World Health Organization (WHO) recommends individual with signs and symptoms that suggest TB infection should seek care within 2–3 weeks of the symptoms initiation [6]. Moreover, studies reported from different settings on presumptive TB patients recommend that the effectiveness of early diagnosis and commencement of the treatment in the prevention of prolonged disease transmission [7–9]. However, considerable proportion of presumptive TB patients are not timely seeking care from health facilities in different countries [7–9]. For instance, community based study reported from Zambia shown that 65.1% of individuals with presumptive TB do not seek care from health facilities for their symptoms [7]. Other study reported from Indonesia also indicated that 11.3% of individuals with presumptive TB do not seek care from any source [8]. Moreover, study reported from Nigeria also shown that large number (75.2%) of individuals with presumptive TB delay health care seeking for more than 30 days after the onset of symptoms [9].

Several reasons of not seeking health care from health facilities have been reported from different settings [7–13]. For example, male sex, urban residence, high income quintile, educated [7], mild symptoms and financial burden [8] are the main reason why patients with presumptive TB are not seeking health care from health facilities. Furthermore, poor knowledge about TB cause and transmission mode, belief in prayers and in house healing [9], unemployment, old age and illiteracy [10] are the main risk factors of not seeking health care. Cost of diagnosis and isolation from family [11]; lack of transportation means [12]; culture or tradition, stigma and witchcraft [13] are also the reasons why individuals with presumptive TB are not seeking health care from health facilities.

Ethiopia is one of the high TB, TB/HIV and MDR-TB burden countries [14] with an estimated prevalence of 108 per 100,000 population in smear-positive drug susceptible TB individuals [15]. In Ethiopia there are several studies that reported health facility related treatment initiation delay among TB patients [16–21]. However, few studies are conducted on the health seeking behavior from health facilities among presumptive TB patients. Moreover, although there are few studies with varied findings on health seeking behavior from health facilities among presumptive TB patients, there is no systematic review and meta-analysis that attempted to summarize the available evidence to support policy making in Ethiopia. Therefore, this review was aimed to estimate the pooled proportions of health care seeking and not seeking behaviors among presumptive TB patients from health facilities, and to summarize the reasons why the patients with presumptive TB are not seeking care from health facilities in Ethiopia.

## Methods

### Searching strategy

**Fig. 1:**
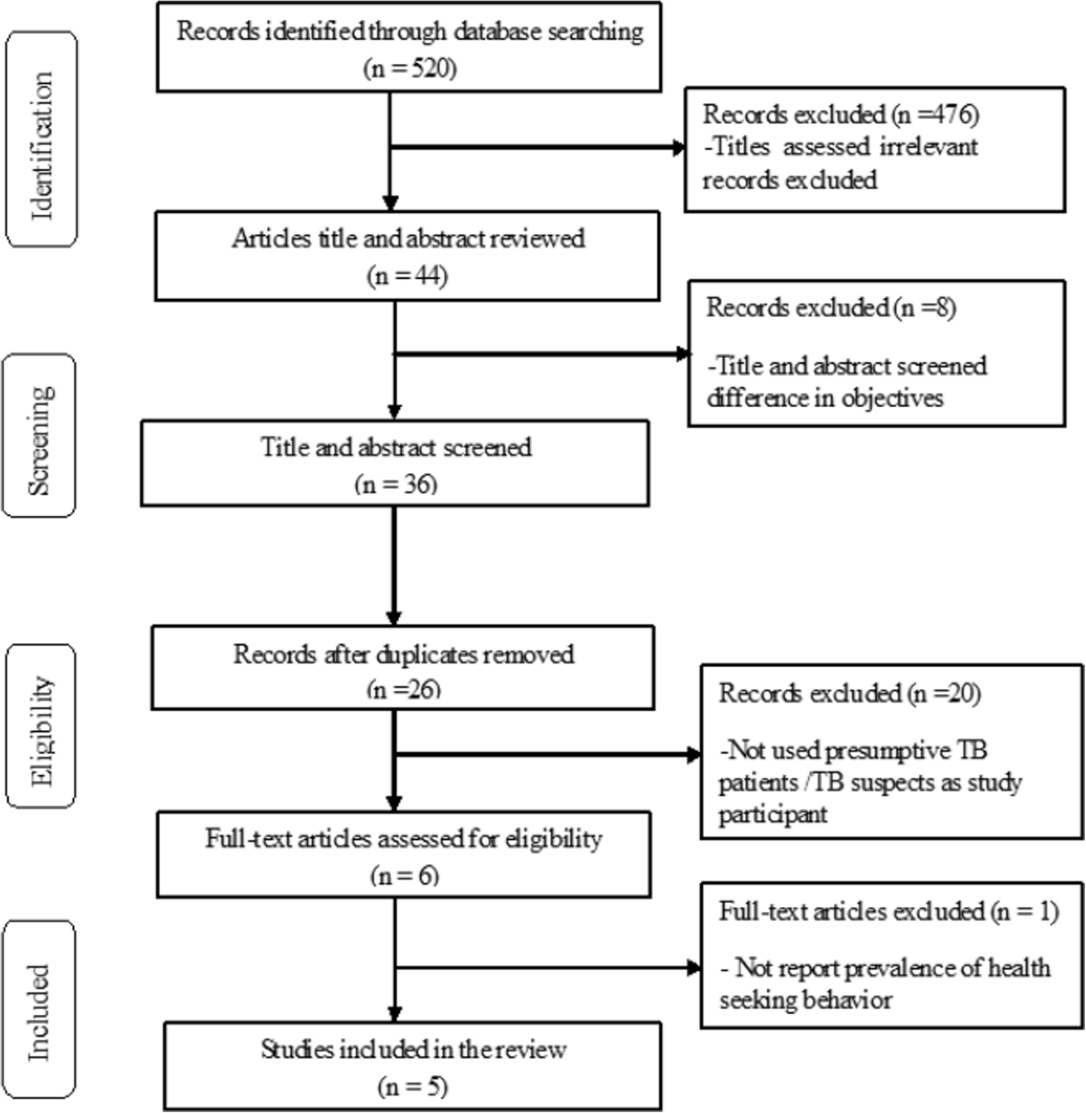
PRISMA flow diagram for article selection and screening [22]

A systematic review and meta-analysis study was conducted to estimate the pooled proportions of health care seeking and not seeking behaviors from health facilities among presumptive TB patients, and to summarize the reasons why the patients are not seeking care from health facilities. PRISMA (Preferred Reporting Items for Systematic Review and Meta-analysis) flow diagram standards (Fig. 1 and PRISMA checklist) were followed during review process. Electronic databases such as PubMed, Google Scholar and Science Direct were searched for studies reported in English language without restricting publication year. The search was conducted from January 1/2019 to March 05/2019. The bibliographies of included articles were also screened for the relevant studies. The search strategy was used by combining the keywords: “health seeking”, “health seeking behavior”, “TB suspects”, “presumptive TB” and “TB treatment delay” both in Medical Subject Heading (MeSH) and free text terms.

### Inclusion and exclusion criteria

Cross-sectional study design that reported proportion of health care seeking behavior among presumptive TB patients were included without restricting publication year (Table 1). In addition, studies conducted both at community and health facility levels were included (Table 1). However, studies that did not report proportion of health care seeking behavior and duplicated were excluded (Fig. 1). Two authors (DFG, HHT) independently conducted search and screened the studies based on study title and abstract at the first step. In the next step both authors were selected full text studies based on eligibility criteria independently. In the case of disagreements, the two author’s disagreement were resolved by the decision of the third author (ZM).

**Table 1:** Characteristics of study included in the review and meta-analysis

Authors	Year	Methodology and Type of study design	Study tool and Sample size	quality score of included studies
Abebe et al. [23]	2009–2010	Community based, cross-sectional study	Structured questionnaire and 476 TB suspects	7/10 (fair)
Engeda et al. [24]	2014–2016	Community based, cross-sectional study	Structured questionnaire and 663 TB suspects	7/10 (fair)
Hiluf et al. [25]	2012–2014	Institutional based, cross sectional survey	Interviewer questionnaire and 242 TB diagnosed women patients	4/10 (poor)
Senbeto et al. [26]	2012–2013	Population based, cross-sectional study	Structured questionnaire and 843 TB suspects	7/10 (fair)
Yimer et al. [27]	2008–2009	Community based, cross-sectional study	Semi-structured questionnaire and 1006 TB suspects	7/10 (fair)

### Study quality assessment

The quality of the included studies were assessed by the modified version of Newcastle-Ottawa quality assessment scale (NOS) [28]. The scale assesses three key points (domains) of a given study: participant selection, comparability of the groups and outcome measurement. Star was assigned for each point of the scale to categorize the studies into good, fair and poor quality based on the NOS criteria [28]. As a result, a good quality study was obtained 3 or 4 stars in participant selection, 1 or 2 stars in comparability of groups, and 2 or 3 stars in outcome assessment. Similarly, a fair quality study was scored 2 stars in participant selection, 1 or 2 stars in comparability of the groups and 2 or 3 stars in outcome assessment. However, a poor quality study was scored 0 or 1 star in participant selection, 0 stars in comparability of the groups and 0 or 1 star in outcome assessment. In case of disparity between the two authors (DFG and HHT) on the quality of the study, the disparity was resolved by the decision of the third author (ZM).

### Study selection

In the first step the articles were identified based on the objectives of the study, and screened by the titles and the abstracts. The articles that were not satisfied the inclusion criteria were excluded at the second stage. After this step, the eligible articles were further screened by reading the full texts and those did not meet the inclusion criteria were excluded. Finally, the eligible full text articles that fulfilled the inclusion criteria were included in the review.

### Data extraction

Data was extracted on Microsoft excel spreadsheet from the included studies. The primary outcome of this study was health care seeking and not seeking behaviors that were measured by interviewer guided questionnaire. Information was extracted on characteristics of participants such as age range, mean of age and sex. Data was also extracted on studies’ characteristics: name of first author, publication year, study setting and sample size. In addition, proportion of health care seeking behavior from health facilities, proportion of not seeking health care from any sources, proportion of seeking health care from inappropriate sources and reasons of not seeking care were extracted from each included studies.

### Statistical analysis

Data analysis was conducted by STATA version 14 (StataCorp, College Station, TX, USA). Random effect model meta-analysis was employed to estimate pooled proportion of health care seeking and not seeking health care behaviors. The heterogeneity among effect sizes of included studies was evaluated using the Q test results with significance difference at *p* < 0.1 and the I^2^ statistic value > 75% [29]. Potential publication bias was assessed by funnel plot. The effects of sample size and publication year on the heterogeneity in the proportions of health care seeking and not seeking health care behaviors were assessed by moment based meta-regression models. We also qualitatively summarized the reasons why the patients are not seeking health care from health facilities, because few studies were reported common risk factors to estimate the pooled risk levels.

## Result

### Included study description

Table 1 shows the characteristics of included studies. Out of 520 records obtained through electronic database search only five studies were met our inclusion criteria and were included to this review. Of the five articles included four [23, 24, 26, 27] were community based cross-sectional studies, while one was institution based study [25]. Studies publication year were ranges from 2008 to 2014. Three articles were reported from Amhara [24, 26, 27], one from Oromia [23] and one from Tigray national regional states [25]. All studies used interviewer guided structured questionnaire to collect data on health seeking behavior and other important variables. The smallest sample size of the included studies was 242 participants [25], while the largest was 1006 [27].

### Health seeking behavior

**Fig. 2:**
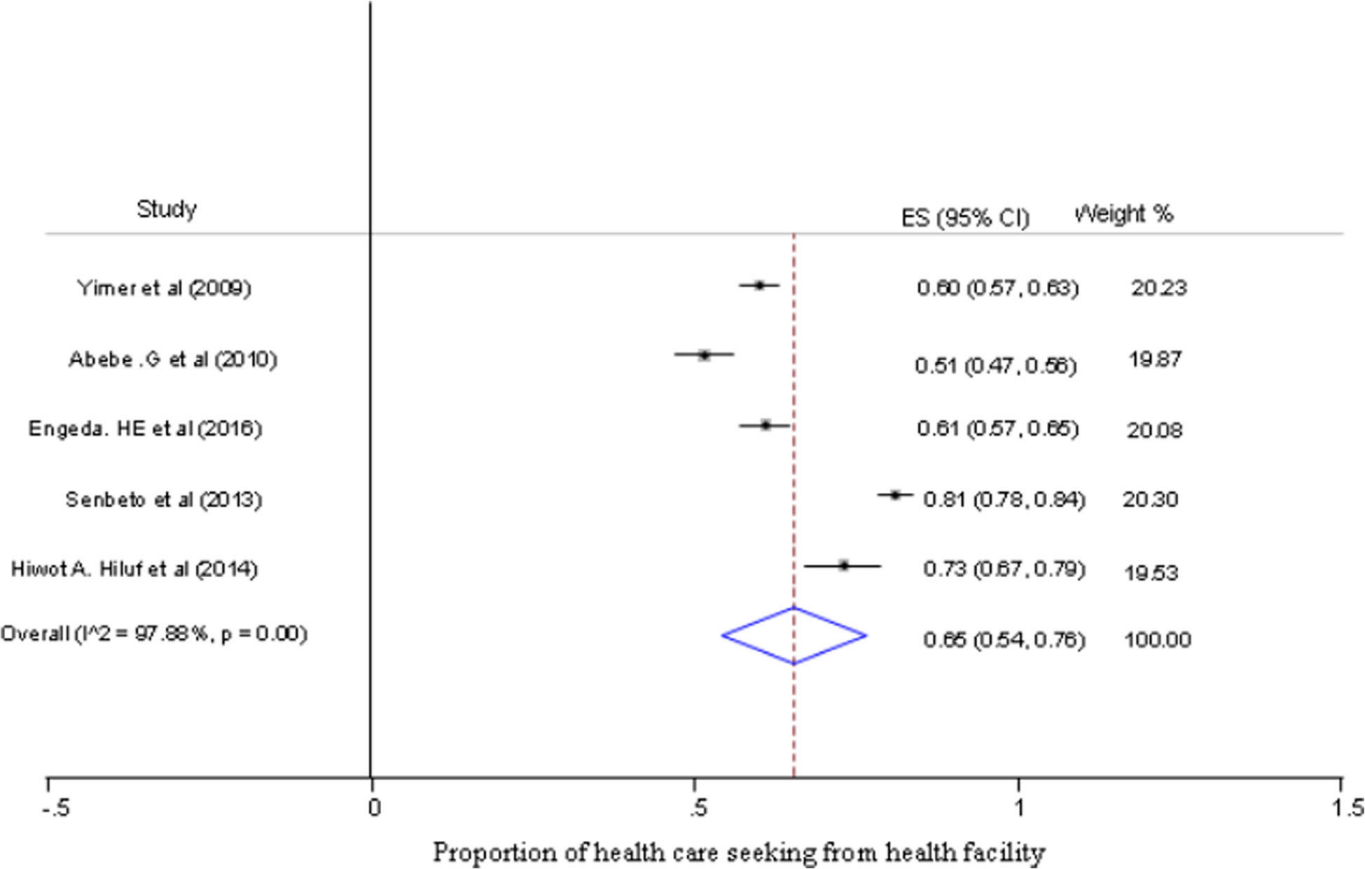
Forest plot of pooled proportion of health care seeking behavior in presumptive TB patients in Ethiopia (Pooled proportion estimated by random-effect model, *ES = effect size)

**Fig. 3:**
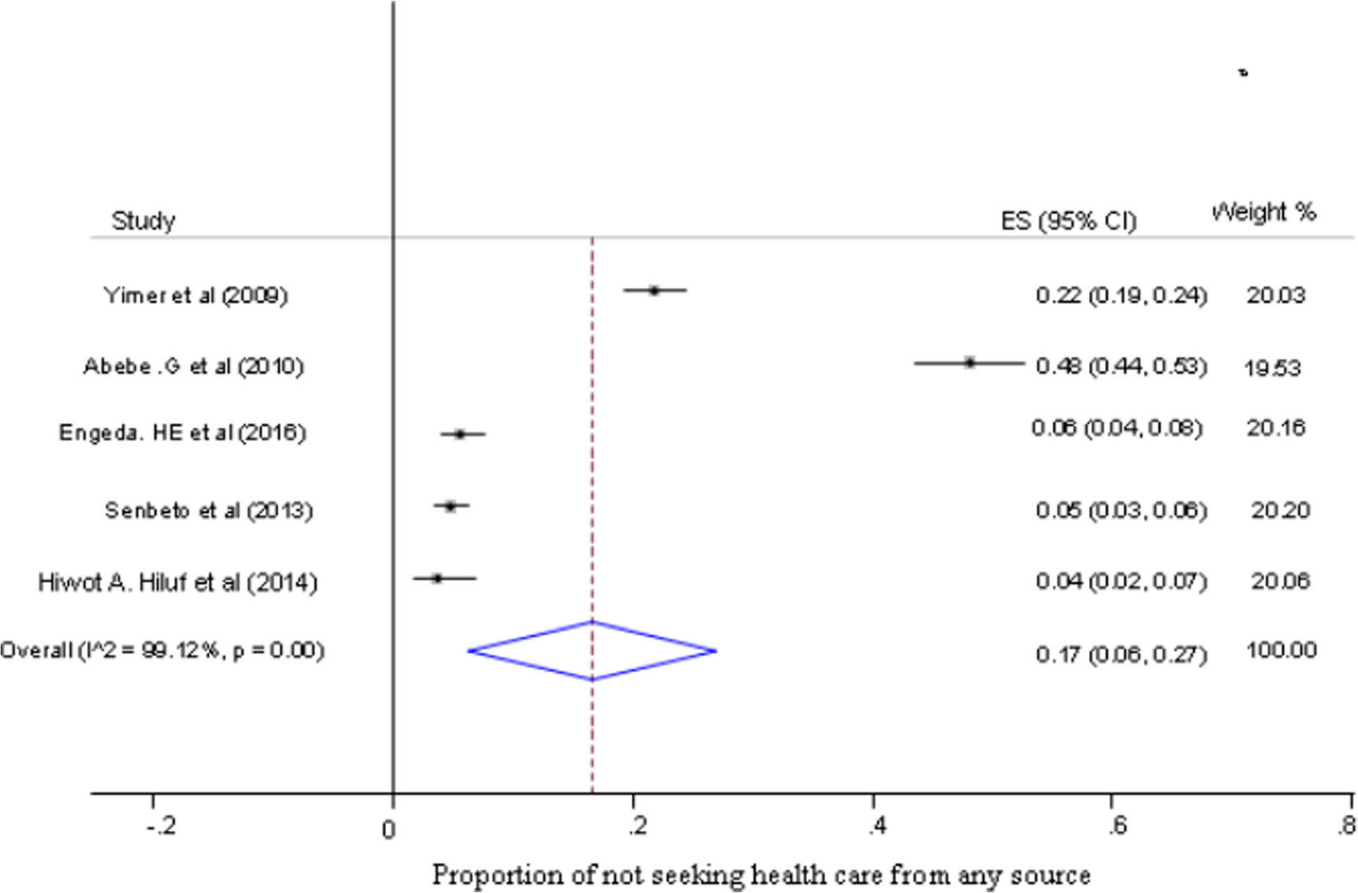
Forest plot of pooled proportion of not seeking health care from any source among presumptive TB patients in Ethiopia (Pooled proportion estimated by random-effect model, *ES = effect size)

**Fig. 4:**
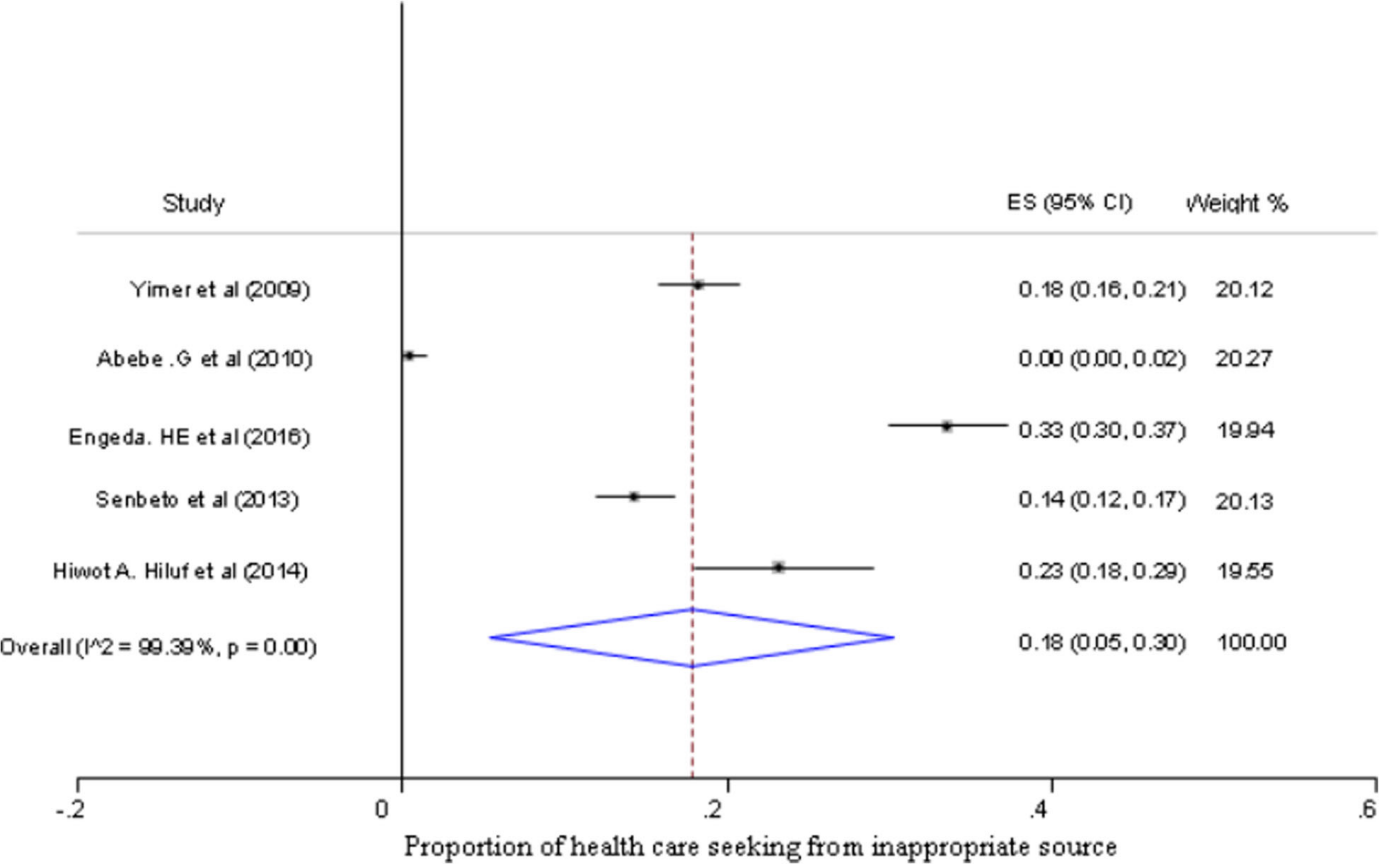
Forest plot of pooled proportion of seeking health care from inappropriate source in presumptive TB patients in Ethiopia (Pooled proportion estimated by random-effect model*,* *ES = effect size*)*

The largest crude proportion of health care seeking behavior from health facilities among individuals with presumptive TB was 81% (95% CI, 78 to 84%), while the smallest was 51% (95% CI; 47 to 56%) (Fig. 2). The estimated pooled proportion of health care seeking behavior among presumptive TB patients in Ethiopia was 65% (95% CI; 53 to 76%) (Fig. 2), while not seeking health care from any sources was 17% (95% CI; 6 to 27%) (Fig. 3). Moreover, the estimated pooled proportion of seeking health care from inappropriate sources was 18% (95% CI; 5 to 30%) (Fig. 4).

We used random effect model because the overall results of Chi-square based Q test and I^2^ statistic (variation in effect sizes attributable to heterogeneity) shown high heterogeneity among results of the studies (Q = 188.43, df = 4, *p* value < 0.001 and I^2^ = 97.88%) for proportion of health care seeking estimation.

### Reason for not seeking care from health facilities

Short duration of cough, absence of previous TB treatment history, lack of money, old age, low education level, lack of job, perceived wellness and use of traditional healing practice were the most frequently reported reasons why patients were not seeking care from health facilities (Table 2). However, male sex, independence in economy, less family size, and being unmarried were factors that promoted health care seeking behavior from health facilities (Table 2).

**Table 2:** Reasons for seeking and not seeking health care from health facilities

Authors	Reasons not seeking care from health facilities	Reasons of seeking care from health facilities
Abebe et al. [23]	Absence of previous TB treatment history	–
Engeda et al. [24]	Old age, less than secondary educational level, lack of job, perceived wellness, absence of previous TB treatment history	–
Hiluf et al. [25]	Age group greater than 55 years,Lack of money, illiteracy	Being unmarried
Senbeto et al. [26]	Low monthly income, use of traditional-healing practices.	Less family size, male sex
Yimer et al. [27]	Short duration of cough, absence of previous TB treatment history	Male sex, independence in economy

-the reasons not reported

### Publication bias

Based on the funnel plot there was a sign of publication bias on the estimated proportions of health care seeking, not seeking and seeking health care from inappropriate sources behaviors (Additional file S1).

### Meta regression analysis

We have assessed the effect of sample size and year of study on heterogeneity between each studies using moment based meta regression model (Table 3).The results shown that both sample size and study year did not significantly predict the heterogeneity of included studies’ effect sizes in health seeking behavior from health facilities, not seeking care at all from any source and seeking care from inappropriate sources (Table 3).

**Table 3:** Meta regression analysis for year of study and sample size as reason of heterogeneity on proportion of health care seeking behavior from health facilities, not seeking health care from any sources and seeking health care from inappropriate sources

Predictive Variable	Health care seeking behavior from Health Facilities				Not seeking health care from any sources				seeking health care from inappropriate sources			
	Unadjusted model		Adjusted model		Unadjusted model		Adjusted model		Unadjusted model		Adjusted model	
	β(95%CI)	P value	β(95%CI)	P value	β(95%CI)	P value	β(95%CI)	P value	β(95%CI)	P value	β(95%CI)	P value
Year of study	14.8(− 152—122.4)	0.75	13(− 68.3—94.8)	0.557	− 33.81(− 152— -4.5)	0.035	−33.6(− 85.9—18.6)	0.109	10.05(− 44.23—64.3)	0.59	−20.38(−26.22—7)	0.2
Sample size	0.68 (0.19—1.17)	0.021	0.7 (0.054—1.5)	0.057	0.12(−0.49—0.74)	0.568	0.0045 (0.496—0.5)	0.97	0.19(−0.22—0.61)	0.23	0.26(−0.18—0.7)	0.12

## Discussion

Not seeking or delay in seeking health care from health facilities for TB disease have a potential risk in prolonged disease transmission and poor treatment outcome [3, 5]. In Ethiopia there are few primary studies that attempt to determine health care seeking behavior in presumptive TB patients. However, there was no review study that tried to summarize the available evidence to support policy making and promote health care seeking behavior. In this systematic review and meta-analysis, data of 3230 presumptive TB patients from five studies were pooled to estimate the proportion of health care seeking and not seeking behaviors from health facilities. In this review, the pooled proportion of seeking health care from health facilities was 65%, while the pooled proportion of not seeking health care from any sources was 17%. Considerable proportion 18% of patients with presumptive TB was seeking health care from inappropriate sources. The most frequently reported reasons why patients with presumptive TB are not seeking health care from health facilities were short duration of cough, being new TB case, lack of money, old age, low education level, lack of jobs, perceived wellness and use of traditional healer.

The pooled proportion of our review indicated that considerable proportion (35%) of patients with presumptive TB was not seeking health care from health facilities. This finding was lower than the findings reported from Zambia in which the proportion of not seeking health care from health facilities was 65.1% [7]. This difference might be due to the discrepancy in TB prevention and control program strength and sociocultural difference between the study populations. For instance, Ethiopia has implemented health extension program that serve the community at home level on basic health care and utilization programs. Health-extension workers in Ethiopia provide health education on communicable disease prevention and seeking health care for their illness. This might be the probable reasons why our pooled proportion of not seeking health care from facilities is lower than the previous report from Zambia [7]. Moreover, the pooled proportion of not seeking health care from any sources in this review was 17% which was higher than the findings reported from Indonesia in which the proportion of not seeking health care is 11.3% in individuals with presumptive TB [8]. This difference most probably due to difference in TB control program implementation quality and sociocultural difference between study settings.

The pooled proportion of seeking health care from inappropriate sources in this review was 18%. This finding was lower than the study reported from Jharkhand in which 30.8% of the patients took self-medication from local shops for their TB symptoms [30]. In addition, a systematic review and meta-analysis conducted in low and middle income countries shown that, seeking health care from informal providers is the key determinant of TB patient delay from seeking health care [31]. This difference might be due to the reasons provided in above paragraph which are discrepancy in sociocultural, strength in TB program implementation and health education provision methods between the countries. Furthermore, the availability of health extension workers who create awareness regarding TB symptoms and free availability of the treatment could have role in seeking health care for TB symptoms in Ethiopia.

Short duration of cough, absence of previous TB treatment history, lack of money, old age, low education level, lack of jobs, perceived wellness and use of traditional healer were the most frequently reported reasons why patients with presumptive TB are not seeking health care from health facilities. This result was in line with the findings of systematic review reported by Storla et al. [32] and Jebamalar et al. [33] in which old age was negatively associated with delay for TB diagnosis. In contrast to our finding, the primary study reported from Zambia indicated that older age groups are more likely seek care than the youngster [7]. In addition, low education level was the reasons why the patients are not seeking health care from health facilities. This result was similar with the systematic review reported by Storla et al. [32] in which low educational level is associated with delay health care seeking in presumptive TB patients. In contrast, the study reported from Zambia shown that highly educated individuals are less likely seek care than uneducated patients with presumptive TB [7].

Tuberculosis is considered as the disease of poverty, because it mainly affects poor and socially disadvantaged people. In this review, economic constraint was among the main reason why the patients are not seeking health care. This finding was similar with the results of primary studies reported from Jordan and India in which economic constraint is the most obstacle for health seeking behavior [12, 33].

Our review also revealed that, male sex, independence in economy, less family size and being unmarried were the main factors that promoted health care seeking behavior from health facilities. This findings are consistent with the results of a systematic review reported by Storla et al. [32]. However, our finding was inconsistent with the study reported by Chanda-Kapata et al. [7] in which males are less likely seek care for TB symptoms than females. Similarly, study reported from Pakistan indicated that females are seek health care for TB diagnosis than males [34]. These differences might be as a result of female population being higher than male in the countries. Furthermore, these difference most probably could be due to social and cultural difference of the studies population and the awareness level difference regarding TB disease and its treatment in the two communities.

The main strength of this review is employing random effect model to address heterogeneity among the studies. However, this review also had some limitation such as inclusion of small number of studies which limited us to conduct sub-group analysis to explore the potential source of heterogeneity, and resulted in wide estimation range of pooled proportions. Promoting research on health care seeking behavior and reasons why patents with presumptive TB are not seeking care from health facility are important to understand the real situation of health care seeking behavior. In addition, the electronic databases searched were limited to PubMed, Google-scholar and Science Direct due to lack of free access to other electronic databases. This might introduce publication bias to this review.

## Conclusion

This review revealed that considerable proportion of patients with presumptive TB are not seeking health care from health facilities in Ethiopia which could leads to prolonged disease transmission and poor treatment outcome. Several and interrelated factors are reported as the main reasons why the patients are not seeking health care from health facilities in Ethiopia. Efforts should be implemented to improve health care seeking behavior from health facilities which could prevent disease transmission and increase treatment success. Health extension program which is one of the key elements of health policy in Ethiopia should promote TB health care seeking behavior in a community. In addition, promoting research on health care seeking behavior among patients with presumptive TB is vital to address evidence gap on this important public health problem.

## Supplementary information

**Additional file 1.** Figures (funnel plots) S1.

**Additional file 2.** Dataset S2.

## Data Availability

All data generated or analyzed during this study were included in this published article as supporting data.

## Abbreviations

HIV: Human Immune Virus
MDR-TB: Multi Drug Resistant Tuberculosis
MeSH: Medical Subject Heading
NOS: Newcastle-Ottawa quality assessment scale
PRISMA: Preferred Reporting Items for Systematic Review and Meta-analysis
TB: Tuberculosis
WHO: World Health Organization
