# Supplementary figures and images for: Health care seeking behavior among presumptive tuberculosis patients in Ethiopia: a systematic review and meta-analysis
Source: BMC Health Serv Res. 2020 May 19;20:445. doi: 10.1186/s12913-020-05284-5 (PMC7238571; doi:10.1186/s12913-020-05284-5)

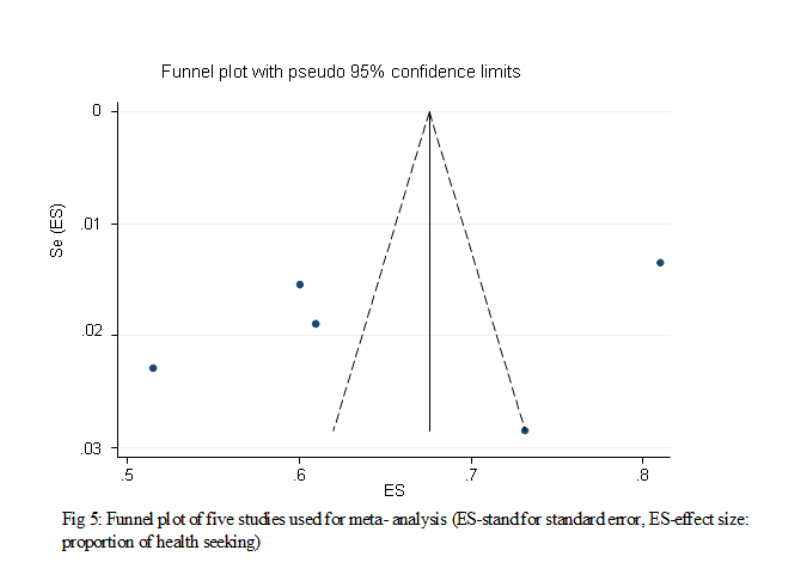

Supplement: Supplementary file 1 — Additional file 1. Figures (funnel plots) S1. [file 12913_2020_5284_MOESM1_ESM.zip › Fig 5 Funnel plotR4.tif]

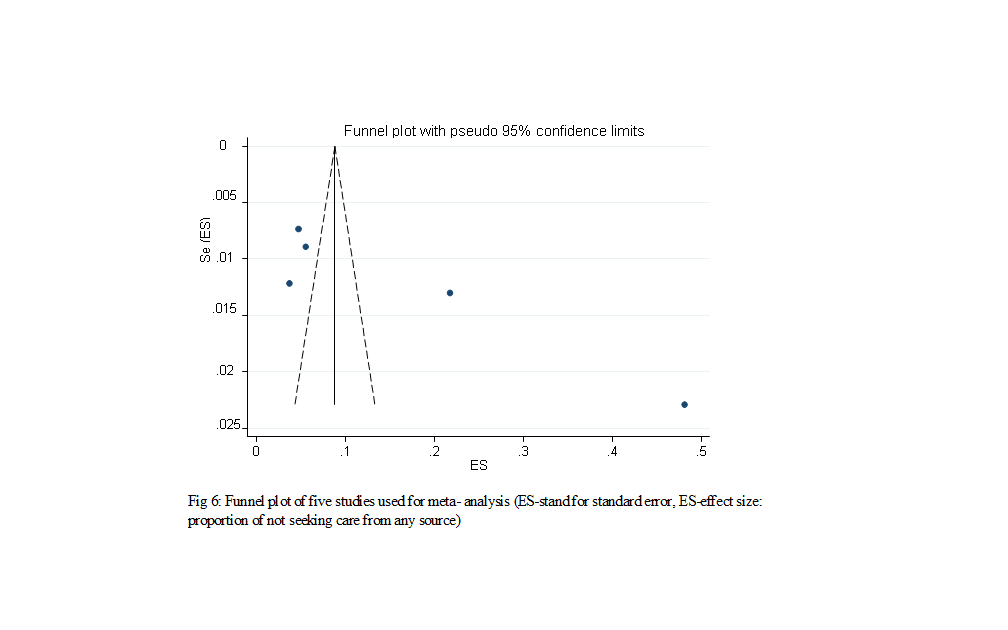

Supplement: Supplementary file 1 — Additional file 1. Figures (funnel plots) S1. [file 12913_2020_5284_MOESM1_ESM.zip › Fig 6 Funnel plotR4.tif]

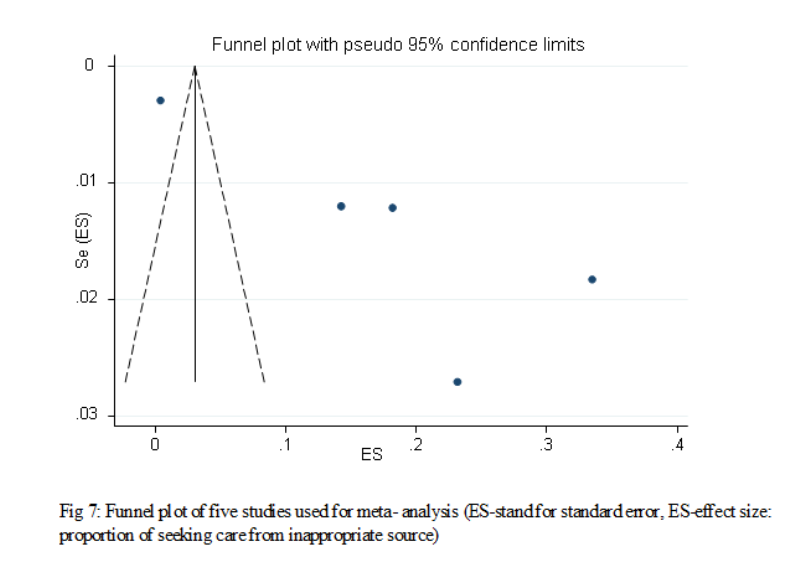

Supplement: Supplementary file 1 — Additional file 1. Figures (funnel plots) S1. [file 12913_2020_5284_MOESM1_ESM.zip › Fig 7 Funnel plotR4.tif]
